# Introduction to the *Neurophotonics* Special Issue “Imaging Brain Metabolism and Neuroenergetics”

**DOI:** 10.1117/1.NPh.12.S2.S22801

**Published:** 2026-01-28

**Authors:** Alberto L. Vazquez, Ghazaleh Ashrafi, Prakash Kara

**Affiliations:** a University of Pittsburgh, Department of Radiology, Pittsburgh, Pennsylvania, USA; b Washington University School of Medicine in St. Louis, Department of Cell Biology and Physiology, St. Louis, Missouri, USA; c University of Minnesota, Medical School, Department of Neuroscience, Minneapolis, Minnesota, USA

## Abstract

The editorial introduces the articles in the Neurophotonics Special Issue on Imaging Brain Metabolism and Neuroenergetics.

The brain consumes about 20% of the body’s metabolic supply and its high energetic cost is evident in the necessary and continuous supply of blood to sustain function. The brain lacks substantial energy reserves, therefore tight regulation between blood supply and neuronal activity is required. Oxygen is the primary energetic substrate and its delivery to tissue is largely dictated by blood flow. Although a multitude of studies have studied this relationship, much remains unknown and optical tools are providing new insights into neuro-metabolism and neuro-vascular regulation with incredible detail. In this *Neurophotonics* special issue, novel tools to examine different aspects of neuro-metabolism regulation using optical methods are discussed. Zhao et al. (doi 10.1117/1.NPh.12.S2.S22805) present a broad array of optical biosensors for metabolic imaging and their application in the study of neurodegeneration, while Kim, Navedo and Tran (doi 10.1117/1.NPh.12.S2.S22809) discuss optical sensors for monitoring signaling pathways, especially voltage and cyclic AMP activity, that can be targeted to different brain cell types. Two new vascular genetic sensors are described with different fluorescence characteristics. Baeza-Lehnert et al. (doi 10.1117/1.NPh.12.S2.S22807) provide a step-by-step protocol for quantification of cytosolic pyruvate in neurons using a fluorescence resonant energy transfer (FRET) sensor. This approach can provide much needed metabolic information in different experimental conditions.

Larios, Sullere, and Gu (doi 10.1117/1.NPh.12.S2.S22810) review known molecular and cellular mechanisms underlying neuro-vascular signaling that likely serve essential metabolic roles. The article highlights key physiological functions for energetic supply and their dysregulation and contribution to neurodegeneration. The study by Giblin et al. (doi 10.1117/1.NPh.12.S2.S22803) examined an important contribution to blood and metabolic supply by measuring changes in vascular oxygen tension during capillary flow disruptions. They found that a subset of stalled capillaries reach extremely low local oxygen, resulting in transient hypoxia in surrounding tissue. While these transient events might not be detrimental to the surrounding tissue, repeated events likely have a detrimental impact. Their findings have significant translational value since transient stalls in capillary flow have been observed at elevated levels in preclinical models of neurological disease, highlighting the need for continuous and uninterrupted of blood supply.

It is becoming evident that neurons expressing nitric oxide synthase (NOS) exert strong vaso-regulatory control. The paper by Lee et al. (doi 10.1117/1.NPh.12.S2.S22802) examined the contribution of NOS neurons to neuro-vascular responses when activated by evoked somatosensory stimulation and found that under light anesthesia they do not exert a strong influence over the initiation of neurovascular responses. These findings suggest that sensory stimulation might engage different populations of neurons with sustained network activity. Next-generation genetic vascular labels that target hepatocytes to make fluorescent albumin as a relatively long-term vascular agent are presented by Knak et al (doi 10.1117/1.NPh.12.S2.S22811). These novel tools can be used to study vascular function and integrity for longer periods of time including longitudinal experiments without the need to continually apply inject vascular labels. Skøtt, Matchkov, and Postnov (doi 10.1117/1.NPh.12.S2.S22804) used label-free laser speckle contrast imaging to examine natural and spontaneously occurring vascular fluctuations at low frequencies (0.05 to to 0.25 Hz) that are prominent during task-free or resting-state experiments and often show neurogenic origin. They found these slow vascular fluctuations repeat about every 80 sec and transiting from arteries to veins in about 0.4 sec.

Lastly, two articles provide important reviews of optical experimentation strategies to rigorously study neuro-vascular and neuro-metabolic events. An accessible and comprehensive guide of two-photon microscopy experiments to study neurovascular function in rodents that include surgical considerations, vessel identification, and novel imaging techniques is presented by Lind, Kucharz, and Cai (doi 10.1117/1.NPh.12.S2.S22808). The paper by Kutozov and Lauritzen (doi 10.1117/1.NPh.12.S2.S22806) highlights the importance of optimizing both spatial and temporal resolution in two-photon microscopy (TPM) experiments. They discuss the impact of light scattering through brain tissue in TPM experiments, as well as the impact of motion and blur arising from the natural movement of cerebral vasculature and the diffusion of tracers, respectively. Altogether this collection presents novel methods, findings, and strategies to continue unraveling cerebral metabolism in the working brain.

